# Fear of cancer recurrence in breast cancer survivors carrying a *BRCA1* or *2* genetic mutation : a cross-sectional study

**DOI:** 10.1186/s13053-024-00285-5

**Published:** 2024-08-27

**Authors:** Alexandra Michel, Michel Dorval, Jocelyne Chiquette, Josée Savard

**Affiliations:** 1 School of Psychology, Université Laval, Québec, Canada; 2 CHU de Québec-Université Laval Research Center, Québec, Canada; 3 Université Laval Cancer Research Center, Centre intégré de cancérologie du CHU de Québec-Université, Laval Hôpital de l’Enfant-Jésus, Québec, 1401 18e Rue G1J 1Z4 Canada; 4 Faculty of Pharmacy, Université Laval, Québec, Canada; 5 Faculty of Medicine, Université Laval, Québec, Canada; 6 CISSS de Chaudière-Appalaches Research Center, Levis, Canada

**Keywords:** Fear of cancer recurrence, Breast cancer, *BRCA1/2* genetic mutation, Prophylactic surgery

## Abstract

**Background:**

Fear of cancer recurrence (FCR) affects virtually all patients who have been treated for cancer, to varying degrees. Breast cancer survivors who carry a *BRCA1* or *BRCA2* gene mutation are at high risk of cancer recurrence. No study has yet assessed FCR specifically in this population.

**Objectives:**

This cross-sectional study, conducted in women who were treated for breast cancer and carrying a *BRCA1/2* mutation, aimed to: (1) assess the mean level of FCR and estimate the proportion of patients with clinical levels of FCR; (2) examine the relationships between FCR and selected psychological variables (e.g., avoidance, intolerance to uncertainty) and quality of life; (3) explore whether FCR levels vary as a function of the past preventive treatment received; and (4) to assess the associations between FCR and the presence of decisional conflict or regret regarding the various preventive options.

**Method:**

Participants were recruited through an e-mail sent to an oncogenetic network mailing list (Réseau ROSE). Participants were asked to complete a battery of questionnaires online assessing FCR and other psychological and quality of life variables.

**Results:**

A total of 89 women completed the survey. Most participants had undergone a preventive mastectomy (62.9%) and a preventive salpingo-oophorectomy (75.3%) at the time of the study. The mean Fear of Cancer Recurrence Inventory-severity score was 16.8, which exceeds the clinical cut-off score of 13, and 70.8% of the participants showed a clinical level of FCR. FCR was significantly associated with higher levels of anxiety and depression, and higher avoidance and intolerance of uncertainty, but not with quality of life. No significant difference was observed on the total FCR score between women who had received preventive surgery (mastectomy and/or salpingo-oophorectomy) and those considering it, and those not considering it. The association was significant between higher FRC scores and greater decisional conflicts and regrets about choosing to undergo preventive surgery.

**Conclusion:**

These data suggest that FCR is a significant problem for breast cancer survivors carrying a *BRCA1/2* genetic mutation, even after undergoing a prophylactic surgery. This highlights the importance of providing these women with specific psychological intervention focusing on FCR.

## Introduction

Fear of cancer recurrence (FCR) is defined as the fear, worry, or concern about the possibility of cancer returning or progressing [1]. FCR affects almost every patient who has been treated for cancer, to varying degrees. Indeed, FCR is one of the most reported concerns by patients [2] and coping with FCR was identified as one of the most common unmet psychosocial needs by patients [3, 4]. Episodic FCR, particularly in the period surrounding medical tests, which subsequently decreases when negative results are communicated, is considered normal. However, in some cases, FCR will become problematic [1]. In fact, among patients with cancer, a meta-analysis by Simard et al. [4] showed that 22–87% (Mean = 49%) had moderate FCR, whereas 0–15% (Mean = 7%) reported a high level. FCR can occur at any time during the care trajectory, at diagnosis, during active treatment, after treatment, or in the palliative care phase [5]. Although other evolutions are possible, high/clinical FCR tends to remain stable over time in a large proportion of patients, even in those with a favorable cancer prognosis [5–7].

Hereditary cancers are due to genetic mutations that significantly increase the risk of developing the disease. Among these, mutations in the *BRCA1* and *BRCA2* genes have a particularly important role in the development of breast cancer. Depending on the study, the lifetime risk of developing breast cancer in *BRCA1/2* mutation carriers ranges from 45 to 80% [8–11], which is much higher than in the general population (13%) [12]. In addition to having an increased risk of developing a first breast cancer, women with a *BRCA1/2* genetic mutation and who have been treated for breast cancer are also at an increased risk of having a local recurrence or a second primary cancer. Indeed, a study by Nilsson et al. [13] showed that the cumulative incidence of local recurrence in *BRCA1/2* mutation carriers was 32% and 9% within 15 years after breast-conserving surgery (e.g., lumpectomy) and total mastectomy, respectively. Bordeleau et al. [14] found that the risk of contralateral cancer was significantly higher 5 to 10 years after first diagnosis in carriers, ranging from 20 to 42%, compared with 5% and 6% in non-carriers. However, the prognosis would be the same as for women with sporadic cancer.

Given that women who have been treated for breast cancer and who carry a *BRCA1/2* gene mutation are at a high risk of recurrence, it is plausible that these women are also more likely to display high levels of FCR. However, no study has yet evaluated FCR in this population specifically. The main goal of this cross-sectional study was therefore to assess the extent to which these women deal with FCR.

Another study goal was to assess the relationship between FCR and other psychological variables and quality of life variables. Depression and anxiety are common in breast cancer survivors [15, 16]. *BRCA1/2* mutation carriers are also particularly likely to experience these psychological difficulties. Indeed, two systematic reviews found higher levels of distress, anxiety, and depression in carriers compared to non-carriers following the announcement of the genetic test result [17, 18]. FCR has also been found to be related to depression and anxiety [19–21]. In addition, FCR is associated with worry, ruminations, intrusive thoughts, the use of maladaptive coping strategies such as avoidance and excessive reassurance-seeking, and impaired functioning and quality of life [1, 22]. It is also related to high intolerance to uncertainty [23, 24]. Theoretical models have identified behavioral and cognitive avoidance, and intolerance to uncertainty as critical etiological factors for FCR [25–27].

Prophylactic interventions, such as total (unilateral or bilateral) mastectomy and salpingo-oophorectomy (i.e., resection of ovaries and fallopian tubes), can significantly reduce the risk of recurrence. The literature shows that bilateral prophylactic mastectomy reduces breast cancer risk by 90–95% [28]. A meta-analysis of women with a *BRCA1/2* genetic mutation found that preventive salpingo-oophorectomy was associated with a strong reduction in ovarian cancer risk, ranging from 71 to 96%, and a 50% reduction in breast cancer risk [29]. Although the risk of developing cancer following preventive surgery is significantly reduced, it is never null. Therefore, women will have to continue dealing with the uncertainty associated with a possible recurrence even after they have undergone a preventive surgery, possibly leading to FCR. However, at present, no study has evaluated FCR in relation to undergone surgeries. One goal of this study was to investigate the links between past and planned surgeries and FCR.

Finally, although preventive surgery is highly effective in reducing breast cancer risk, the decision-making process remains difficult for many women because of the physical and psychological consequences of these interventions [30]. Overall, satisfaction with the decision to have undergone preventive surgery is high, although some women express regret about their decision and dissatisfaction with their appearance [31, 32]. Regrets are related to a greater number of complications and a higher level of emotional distress [31]. However, no studies have assessed the levels of decisional conflict and regret associated with FCR.

This cross-sectional study aimed at: (1) assessing the mean level of FCR and estimating the proportion of patients with a clinical level of FCR overall and in various subgroups of patients (e.g., *BRCA1* vs. *BRCA2*); (2) evaluating the relationship between FCR and several psychological variables (i.e., depression, anxiety avoidance, intolerance to uncertainty) and quality of life; (3) comparing FCR levels as a function of the past preventive treatment received; and (4) evaluating the relationship between FCR and the presence of decisional conflict or regret regarding the different preventive options.

It was postulated that the average FCR would correspond to a clinical level of FCR (score of 13 or higher on the severity subscale of the Fear of Cancer Recurrence Inventory; FCRI-S). Since the risk of recurrence is higher in carriers of the *BRCA1* than *BRCA2* gene mutation [33], it was expected that the first group would have higher FCR. In addition, it was hypothesized that women younger than 50 years old would have a greater FCR level, given the higher cancer risk in younger carriers [34]. Based on available evidence, it was also expected that women with higher FCR levels would show more severe anxiety and depressive symptoms and poorer quality of life, as well as higher levels of intolerance to uncertainty and greater use of avoidance as a coping strategy. Further, it was predicted that women who had undergone a preventive surgery would have lower FCR levels than those considering it and those not considering it. Finally, with regard to the fourth objective, it was expected that women with higher levels of FCR would have greater levels of decisional conflict and regrets regarding preventive options, thus possibly reflecting some disappointment in the limited effects that preventive surgery had on the FCR level experienced.

## Methods

### Participants

To be eligible for this study, participants had to meet the following criteria: (a) had been treated for non-metastatic breast or ovarian cancer; (b) carry a *BRCA1* or *BRCA2* mutation; (c) be between 18 and 80 years old; and (d) be able to read and understand French. Participants were excluded if they were on active cancer treatment other than adjuvant hormone therapy. Initially, women treated for ovarian cancer were included in this project. However, due to their under-representation in the sample (*n* = 5), their data were not analyzed.

### Procedure

Participants were recruited in the province of Quebec, Canada, between October 9, 2020 and May 10, 2021 by sending an announcement by email using the distribution list of the ROSE (Ressources en oncogénétique pour le soutien et l’éducation; Oncogenetic Resources for Support and Education [35]) network, which reaches nearly 3,000 individuals including patients, family members and health professionals. Individuals on this list had already consented to be solicited to participate in research projects.

Women interested in participating were invited to complete a battery of questionnaires online through the REDCap interface using a web link provided in the announcement. They were first directed to the consent form describing the project in detail, the involvement required and the benefits and risks of taking part in this project. After they gave their electronic consent, they were directed to the questionnaires. The questionnaires included self-report measures assessing FCR, as well as various psychological symptoms and possible correlates (e.g., intolerance of uncertainty). Participants were invited to contact the graduate student in charge of the research project (AM) if they had any questions or needed clarification regarding their participation. The study was approved by the ethics committee of the CHU de Québec-Université Laval (#2022–5908).

### Measures

A sociodemographic and medical questionnaire was developed to collect information such as age, education level, marital status, employment and income. It also gathers information on certain cancer characteristics (e.g., date of diagnosis), time interval since the genetic test result, family history of cancer, history of preventive and curative cancer treatments, and presence of any other medical condition.

**Fear of Cancer Recurrence Inventory** (FCRI; dependent variable). The FCRI measures multidimensional aspects of FCR over the last month. It is composed of 42 items divided into seven subscales: [1] Triggers (e.g., *Physical examinations (e.g.*,* annual check-up*,* blood tests*,* X-rays) make me think about the possibility of cancer recurrence*) ; [2] Severity (e.g., *I am worried or anxious about the possibility of cancer recurrence*); [3] Psychological distress (e.g., *When I think about the possibility of a cancer recurrence*,* I feel sadness*,* discouragement or disappointment*); [4] Coping strategies (e.g., *When I think about the possibility of a cancer recurrence*,* I try to distract myself*); [5] Functioning impairments (e.g., *Fearing or thinking about the possibility of cancer recurrence disrupts my work or everyday activities*); [6] Self-criticism (e.g., *I think that I worry more about the possibility of cancer recurrence than other people who have been diagnosed of cancer*); and [7] Reassurance (e.g., *I call my doctor or other health professional (to reassure myself)*). Items are rated on a 5-point Likert scale (0 = never/not at all; 4 = all the time/a lot). A score of 13 or more on the severity subscale (FCRI-S) indicates a clinical level of FCR [36, 37]. The FCRI was developed in French and has excellent psychometric properties, including a good internal consistency with a Cronbach’s α of 0.95 [36]. It includes one item that is reverse-scored (item 13). The FCRI total (FCRI-T) and FCRI-S scores were both used in this study; they are the most frequently used in the current FCR literature.

**Hospital Anxiety and Depression Scale** (HADS). The HADS evaluates anxiety and depressive symptoms over the past week [38, 39]. The HADS contains 14 items separated into two subscales, each containing 7 items (Anxiety or HADS-A; Depression or HADS-D). It does not include any somatic items that could be confused with symptoms of a medical condition. Items are scored on a 0 to 3 scale, and a score of 7 or more on one subscale suggests the presence of a clinical level of that symptom. This tool has good internal consistency (α = 0.89) and good test-retest reliability over a period of 6 months (*r* = 0.7; 38).

**EORTC Quality of Life Questionnaire** (QLQ-C30). The Global health status/Quality of life subscale of the QLQ-30 was used to assess participants’ quality of life [40]. It is composed of two items that are scored on a 7-point Likert scale ranging from “1” (very poor) to “7” (excellent). Scores are transformed to range from 0 to 100. This questionnaire has been validated and translated into French by the authors of the original English version. The complete questionnaire has good psychometric qualities, including a good internal consistency (α ≥ 0.70) and correlations of 0.40 or more between all items and their respective scales [40].

**Intolerance of Uncertainty Scale** (IUS). The IUS is used to measure emotional, cognitive, and behavioral reactions to ambiguous situations, the implications of uncertainty, and attempts to control the future [41]. The instrument consists of 27 items evaluated on a 5-point Likert scale ranging from “1” (not at all consistent) to “5” (completely consistent). The level of intolerance of uncertainty is calculated by summing all items. The instrument showed good internal consistency (α = 0.91) and test-retest reliability (*r* = 0.74) at 5 weeks. The English version was shown to be reliable and has been widely used in oncology [42–44].

**Impact of Event Scale** (IES). The IES provides a measure of symptoms related to a specific traumatic experience which is cancer for the current study [45, 46]. The tool comprises three subscales: avoidance, intrusions and hyperarousal, for a total of 22 items. For each item, respondents are asked to indicate the extent to which they had experienced these difficulties in the past seven days. Answers are rated on a Likert scale ranging from “0” (not at all) to “4” (extremely). The IES has a good internal consistency for its three subscales and the total score (α = 0.81–0.93), and a good test-retest reliability over a period of six months (*r* = 0.71–0.76; 45).

**Decisional Conflict Scale** (DCS). The DCS is used to assess the level of decisional conflict experienced by patients regarding the choice of preventive surgery (mastectomy or salpingo-oophorectomy) [47, 48]. This scale measures patients’ feelings of being informed, clarity about their personal values related to the benefits and harms of their decision, perceived decision support, certainty about making a decision about their health, and personal perception of the decision made (e.g., *I am clear about the best choice for me; I am clear about which is more important to me*,* the benefits or the risks and side effects*). It consists of 16 items that are assessed on a 5-point Likert scale ranging from “1” (strongly agree) to “5” (strongly disagree). Scores are transformed to yield a score from 0 to 100, a higher score indicating a greater decisional conflict. This scale has been validated in the context of various medical and health decision situations and has good psychometric qualities, i.e., a test-retest reliability coefficient of 0.81 over an interval of two weeks and an internal consistency coefficient ranging from 0.78 to 0.92 [48].

**Decisional Regret Scale** (DRS). The DRS is used to estimate the patient’s perceived level of regret regarding the decision that was made in choosing preventive surgery or not (e.g., *It was the right decision; I would go for the same choice if I had to do it over again;* 49). It consists of five items and respondents must indicate their agreement on a Likert scale ranging from “1” (strongly agree) to “5” (strongly disagree). The overall score is expressed on a scale of 0 to 100. This tool has good psychometric qualities with a Cronbach’s alpha ranging from 0.81 to 0.92 [49].

### Statistical analyses

Statistical analyses were performed with SPSS software version 26 [50] using a two-sided 5% alpha level. Descriptive statistics (frequencies, means) were first obtained on sociodemographic and medical variables (e.g., age, type of mutation) to characterize the sample. For the first objective, the average FCRI-T score and subscales scores were calculated. The proportion of patients (%) with a clinical level of FCR was also assessed (score of 13 or higher on the FCRI-S; 37). Multivariable linear models were also used to compare FCR scores between different subgroups of patients, that is: *BRCA2* vs. *BRCA1* mutation; women with vs. without a first-degree relative who had cancer; women with vs. without at least one child, and women 50 years old and less vs. more than 50 years old. For age, the sample was divided into two subgroups based on the median age obtained, which was 51 years old. For the second goal, correlations were performed to assess the relationships between FCR and selected psychological variables (e.g., depression, avoidance, intolerance to uncertainty) and quality of life. To investigate the third objective of exploring the extent to which FCR varied as a function of the past preventive treatment received, ANOVAs were performed on the mean FCRI-T and FCRI-S subscale scores, which compared women who have had at least one preventive surgery (mastectomy and/or salpingo-oophorectomy; *n* = 78) with those who were considering it (*n* = 7) and those who were not considering having such surgery (*n* = 4). These analyses should be considered exploratory given the small number of observations in two cells. Two additional ANOVAs were conducted to compare the level of FCR (FCRI-T and FCRI-S) among women who had undergone a contralateral mastectomy (*n* = 11) or salpingo-oophorectomy only (*n* = 22), with those who had had both surgeries (*n* = 45), and those who had not (*n* = 11). A correlation analysis was also performed to assess the relationship between FCRI scores (FCRI-T and FCRI-S) and the time elapsed since the most recent preventive surgery. When more than one surgery had been undergone, the date of the most recent surgery was used. Finally, for the fourth objective, correlations were performed to assess the relationship between FCRI scores (FCRI-T and FCRI-S) and decisional conflict and regret variables.

## Results

### Descriptive statistics

A total of 81 participants completed all questionnaires and 8 participants partially completed them, for a total of 89 participants. All available data were used in the analyses. The sociodemographic data and medical characteristics of the 89 participants are presented in Table 1. Patients were on average 53.5 years old (range: 26–78). Most participants were married or in a common-law relationship (69.7%) and had a high level of education (47.2% had a university degree). Among participants, 48.3% and 53.9% were carriers of the *BRCA1* and *BRCA2* gene mutation, respectively. In addition, 62.9% of the women had undergone a preventive mastectomy while 75.3% had undergone a preventive salpingo-oophorectomy. Time since the most recent cancer diagnosis and the most recent preventive surgery was 95.9 months (8.0 years; range: 1-420 months) and 70.0 months (5.8 years; range: 1-231 months), respectively.

**Table 1:** Participants’ demographic and medical characteristics (*N* = 89)

	M (SD)	*n* (%)
Age (years)	53.5 (11.5)	
Marital status		
Married/Cohabitating		62 (69.7)
Separated/Divorced		22 (24.7)
Single		4 (4.5)
Widowed		1 (1.1)
Education completed		
Primary school		3 (3.4)
High school		17 (19.1)
College		26 (29.2)
University		42 (47.2)
Other		1 (1.1)
Occupation		
Full time work		53 (59.6)
Part-time work		7 (7.9)
Sick leave		3 (3.4)
Unemployed/looking for work		1 (1.1)
Retired		22 (24.7)
Unpaid family work		2 (2.2)
Other		1 (1.1)
Family income		
$40 000 and less		8 (9.0)
$40 001–60 000		14 (15.7)
$60 001–80 000		17 (19.1)
$80 001- 100 000		6 (6.7)
$100 001- 120 000		17 (19.1)
$120 001 and more		20 (22.5)
I don’t know or refuse to answer		7 (7.9)
Number of months since the most recent cancer diagnosis	95.9 (78.5)	
Number of months since the most recent preventive surgery	70.0 (53.9)	
Genetic mutation*		
BRCA 1		43 (48.3)
BRCA 2		48 (53.9)
Knowledge of the mutation at the time of cancer diagnosis		
Yes		16 (18.0)
No		73 (82.0)
Preventive mastectomy		
Surgery performed		56 (62.9)
Surgery considered and planned		2 (2.2)
Surgery considered, but not yet planned		8 (9.0)
Surgery not performed and not considered		23 (25.8)
Preventive mastectomy performed		
Unilateral		13 (23.2)
Bilateral		43 (76.8)
Breast reconstruction		
Yes		51 (58.2)
No		38 (41.8)
Time of reconstruction		
During mastectomy (immediate reconstruction)		37 (72.5)
After mastectomy (delayed reconstruction)		14 (27.5)
Preventive salpingo-oophorectomy		
Surgery performed		67 (75.3)
Surgery considered and planned		6 (6.7)
Surgery considered, but not yet planned		10 (11.2)
Surgery not performed and not considered		6 (6.7)
Number of first-degree relatives who have developed cancer		
0		20 (22.5)
1		34 (38.2)
2		17 (19.1)
3 and more		18 (20.2)
Number of second-degree relatives who have developed cancer		
0		6 (6.7)
1		12 (13.5)
2		10 (11.2)
3 and more		61 (68.6)

*The sum of these percentages exceeds 100% because some patients (*n* = 2) had both mutations

### FCR levels

On average, participants obtained a mean FCRI-T score of 68.9, and a score of 16.8 on the FCRI-S, which is above the clinical threshold (score of 13 or higher; 37). In addition, 70.8% of the participants showed a clinical level of FCR. Table 2 shows the mean scores obtained on each FCRI subscale.

**Table 2:** Mean (SD) scores obtained on FCRI total score and each subscale (*N* = 89)

		M (SD)
FCRI total score (FCRI-T; 0-168)		68.9 (23.7)
FCRI subscales		
	Triggers (0–32)	17.5 (7.3)
	Severity (FCRI-S; 0–36)	16.8 (6.3)
	Psychological distress (0–16)	6.4 (3.9)
	Functioning impairments (0–24)	3.5 (3.9)
	Insight (0–12)	1.1 (2.0)
	Reassurance (0–12)	3.2 (2.1)
	Coping strategies (0–36)	20.5 (6.9)

### FCR levels by participants’ subgroups

Results of multivariable linear models (see Table 3) showed no significant difference on FCRI scores between mutation types *BRCA1* and *BRCA2* and whether women did or did not have at least one first-degree relative who had cancer. No significant difference was also found on whether women did or did not have children. However, younger women (50 years and younger) showed significantly higher scores than older women (more than 50 years) on the FCRI-T score (β = 14.189, *p* < 0.01), as well as on the *severity* (FCRI-S; β = 4.132, *p* < 0.01), *psychological distress* (β = 2.769, *p* < 0.01) and *functioning impairments* (β = 2.515, *p* < 0.01) subscales. No age differences were found on other FCRI subscales. Moreover, although this difference was not significant (*p* = 0.053), women having at least one child tended to have lower FCR-related *psychological distress* than those without children.

**Table 3:** Results of the multivariable linear models comparing various subgroups on FCRI total and subscales scores (*N* = 89)

FCRI factors	Variables	F	*p*	Partial Eta-Squared	β
Total					
	BRCA 2	0.064	0.801	0.001	1.291
	Age	7.596	0.007*	0.084	14.189
	First degree relatives with cancer	0.271	0.604	0.003	3.212
	With at least 1 child	0.563	0.455	0.007	-5.103
Triggers					
	BRCA 2	0.002	0.962	0.000	0.079
	Age	2.476	0.119	0.029	2.587
	First degree relatives with cancer	0.052	0.820	0.001	-0.450
	With at least 1 child	0.530	0.469	0.006	1.581
Severity					
	BRCA 2	0.562	0.456	0.007	1.006
	Age	9.335	0.003*	0.101	4.132
	First degree relatives with cancer	0.552	0.460	0.007	1.204
	With at least 1 child	0.012	0.912	0.000	0.197
Psychological distress					
	BRCA 2	0.222	0.639	0.003	0.377
	Age	11.807	0.001*	0.125	2.769
	First degree relatives with cancer	1.934	0.168	0.023	1.343
	With at least 1 child	3.839	0.053	0.044	-2.086
Functioning impairments					
	BRCA 2	0.161	0.689	0.002	0.329
	Age	9.259	0.003	0.100	2.515
	First degree relatives with cancer	0.048	0.827	0.001	-0.217
	With at least 1 child	0.074	0.786	0.001	-0.298
Insight					
	BRCA 2	0.442	0.508	0.005	0.293
	Age	0.426	0.516	0.005	0.291
	First degree relatives with cancer	0.063	0.802	0.001	-0.134
	With at least 1 child	1.097	0.298	0.013	-0.616
Reassurance					
	BRCA 2	0.273	0.603	0.003	-0.244
	Age	1.490	0.226	0.018	0.574
	First degree relatives with cancer	1.012	0.317	0.012	0.566
	With at least 1 child	0.300	0.585	0.004	-0.340
Coping strategies					
	BRCA 2	0.128	0.721	0.002	-0.550
	Age	0.731	0.395	0.009	1.323
	First degree relatives with cancer	0.235	0.629	0.003	0.899
	With at least 1 child	3.000	0.087	0.035	-3.541

Note FRCI, Fear of Cancer Recurrence Inventory; BRCA2, Comparison of carriers of the BRCA2 gene mutation vs. those with the BRCA1 (this comparison excludes women with both mutations); Age, Comparison of women ≤ 50 years of age vs. women > 50; First degree relatives with cancer, Comparison of women with a first-degree relative who had cancer vs. those without; With at least 1 child: Comparison of women with at least 1 child vs. those without children. *p < 0.01

### Relationship of FCR with psychological variables and quality of life

Significant associations were found between the FCRI-T score and anxiety (*r* = 0.694, *p* < 0.01), depression (*r* = 0.423, *p* < 0.01) and intolerance to uncertainty (*r* = 0.430, *p* < 0.01; see Table 4). FCR was also significantly associated with the IES total score (*r* = 0.551, *p* < 0.01) and all of its subscales, i.e., avoidance (*r* = 0.365, *p* < 0.01), intrusions (*r* = 0.560, *p* < 0.01) and hyperarousal (*r* = 0.375, *p* < 0.01). All of these correlations were of a medium to large magnitude. However, the negative association between the FCRI-T score and quality of life did not reach significance (*r* = -0.171, *p* = 0.128).

**Table 4:** Correlations (Pearson’s) of FCR with psychological variables and quality of life

	*N*	M	SD	1	2	3	4	5	6	7	8	9
1. Fear of cancer recurrence (FCRI)	89	68.9	23.7	1								
2. Anxiety (HADS-A)	88	6.5	4.1	0.694**	1							
3. Depression (HADS-D)	88	2.5	2.9	0.423**	0.504**	1						
4. Intolerance to uncertainty (IUS)	85	49.6	17.7	0.430**	0.476**	0.377**	1					
5. Quality of life (QLQ-C30)	81	73.0	18.1	-0.171	-0.387**	-0.546**	-0.262*	1				
6. Cancer impact (IES-Total)	81	20.8	15.0	0.551**	0.542**	0.350**	0.602**	-0.257*	1			
7. Avoidance (IES-A)	81	8.1	5.7	0.365**	0.341**	0.339**	0.395**	-0.060	0.648**	1		
8. Intrusions (IES-I)	81	8.7	7.6	0.560**	0.518**	0.241**	0.548**	-0.222*	0.915**	0.344**	1	
9. Hyperarousal (IES-H)	81	4.0	5.0	0.375**	0.443**	0.293**	0.515*	-0.361**	0.855**	0.268*	0.819**	1

*p < 0.05; **p < 0.01. Note FCR, fear of cancer recurrence; FRCI, Fear of Cancer Recurrence Inventory; HADS-A, anxiety subscale of the Hospital Anxiety and Depression Scale; HADS-D, depression subscale of the Hospital Anxiety and Depression Scale; IUS, Intolerance of Uncertainty Scale; QLQ-C30, EORTC Quality of Life Questionnaire; IES, Impact of Event Scale; IES-A, Avoidance subscale of the Impact of Event Scale; IES-!, Intrusion subscale of the Impact of Event Scale; IES-H, Hyperarousal subscale of the Impact of Event Scale

### Relationship between FCR level and past preventive treatment received

Results from the ANOVAs that compared women who underwent preventive mastectomy only (*n* = 11), salpingo-oophorectomy only (*n* = 22), both procedures (*n* = 45), and those who received no procedure (*n* = 11) showed no significant between-groups difference on the FCRI-T score, *F* [3, 84] = 0.256, *p* = 0.857, and on the FCRI-S score, *F* [3, 84] = 0.049, *p* = 0.986 (see Table 5). Also, no significant association was found between the FCR level and time since the most recent preventive surgery: FCRI-T score, *r* = -0.166, *p* = 0.147; FCRI-S score, *r* = -0.214, *p* = 0.060.

**Table 5:** Mean (SD) FCRI total score and FCRI severity subscale by preventive surgery undergone

Groups	*n*	FCRI-T	FCRI-S
Preventive mastectomy only	11	65.18 (16.17)	16.09 (5.47)
Preventive salpingo-oophorectomy only	22	70.32 (24.14)	16.95 (6.67)
Both procedures	45	70.18 (25.46)	16.78 (6.75)
No procedure	11	64.91 (23.55)	16.91 (4.97)

Note FRCI-T, Fear of Cancer Recurrence Inventory total score; FRCI-S, Severity subscale of the Fear of Cancer Recurrence Inventory

The ANOVAs that compared women who had undergone preventive surgery (mastectomy and/or salpingo-oophorectomy; *n* = 78), with those who were considering surgery but had not undergone it yet (*n* = 7), and those who were not considering preventive surgery (*n* = 4; see Table 6), showed no significant between-groups differences on FCRI-T scores, *F* [2, 85] = 2.248, *p* = 0.112, and FCRI-S scores, *F* [2, 85] = 1.221, *p* = 0.300. However, the mean FCRI scores were much lower in women not considering preventive surgery compared to the other two groups.

**Table 6:** Mean (SD) FCRI total score and FCRI severity subscale of women having undergone or not preventive surgery

Groups	*n*	FCRI-T	FCRI-S
Underwent preventive surgery *(mastectomy and/or salpingo-oophorectomy)*	78	69.51 (23.80)	16.73 (6.49)
Considering it but have not yet undergone it	7	75.71 (21.91)	19.14 (4.85)
Not considering preventive surgery	4	46.00 (11.80)	13.00 (1.83)

Note FRCI-T, Fear of Cancer Recurrence Inventory total score; FRCI-S, Severity subscale of the Fear of Cancer Recurrence Inventory

### Relationship between FCR and the presence of decisional conflict or decisional regret

Correlational analyses showed a significant positive association between the FCRI-T score and the presence of decisional conflicts (*r* = 0.235, *p* < 0.05) and regrets (*r* = 0.337, *p* < 0.01). The association was also significant between higher FCRI-S score and greater decisional conflicts (*r* = 0.287, *p* < 0.01) and regrets (*r* = 0.335, *p* < 0.01). These correlation coefficients were of a small to medium magnitude.

## Discussion

Overall, the present study revealed that FCR is highly prevalent in women with breast cancer who carry a *BRCA1/2* mutation. This may appear surprising given that the majority of the study sample had undergone preventive surgery. Bearing in mind the cross-sectional nature of this study, these findings suggest that FCR remains elevated following a preventive surgery, although the actual risk of recurrence following such surgery is significantly reduced. Results also showed significant associations between FCR and various psychological variables, including anxiety, depression, intolerance of uncertainty and avoidance, as well as with decisional conflicts and regrets.

The first objective of the study was to estimate the average FCR level in the total sample and in various subgroups. To our knowledge, this is the first study assessing FCR in breast cancer survivors with a *BRCA1/2* mutation. As hypothesized, results showed an average severity of FCR (FCRI-S; M = 16.8) that fell within the clinical range (score of 13 or higher) and more than two thirds of the participants (70.8%) had a clinical level of FCR. This rate is higher than those obtained in previous studies of patients treated for cancer (e.g., breast, lung, pancreatic, endometrial) also using the FCRI-S, which ranged from 53.1 to 60.1% [51]. These results indicate that FCR is a serious psychological issue in BRCA1/2 carriers who have had a cancer history, even among those whose last cancer was diagnosed several years ago. This may be due to the fact that women with a genetic mutation are well aware of their higher risk of recurrence. In fact, a study by McGinty, Goldenberg and Jacobsen [52] reported that a greater perception of breast cancer risk (vulnerability and severity) correlated with a greater FCR level.

Consistently with the current literature, and as hypothesized, younger women had a higher level of FCR (FCRI-T score) than older women. A systematic review by Crist and Grunfeld [5] revealed that younger age was associated in almost every study with higher FCR for different cancer types, including breast cancer [53–55]. This may be due in part to the greater financial impact that cancer may have on young adults with less financial stability, and the fact that, at a younger age, cancer and its treatment are more likely to interfere significantly with the achievement of life goals. This would be particularly true in *BRCA1/2* mutation carriers who develop cancer at a younger age as compared to sporadic cancer cases [9], thus possibly increasing their fear of having another cancer at a young age. However, no significant differences on FCR levels were found whether participants were a *BRCA1* or a *BRCA2* mutation carrier, whether they had children or not and whether they had a first-degree relative who was treated for cancer or not. *BRCA1* mutation carriers have an actual greater cancer risk than *BRCA2* mutation carriers [33, 56]. This result could be due to a lack of information and knowledge about the differential risk but also to the more prominent role of perceived cancer risk, which may differ from the actual risk [52]. Regarding the absence of differences for those having or not having a first-degree relative treated for cancer, again, it suggests that the individual’s own perceived risk of cancer recurrence plays a greater role than having a family history of cancer. Finally, the absence of differences on whether participants had at least one child may be due to the fact that we did not collect information on their children’s’ age. FCR is likely to be greater in mothers of younger children given the greater possible impact a cancer recurrence may have on children’s psychological well-being [57]. The mean age of the sample was also fairly elevated (53.5 years old), hence most mothers probably had adult children. The influence of having young children thus remains to be investigated in the future.

The second objective was to assess the relationship between FCR and several psychological variables. Findings showed that FCR was significantly associated with higher levels of anxiety and depression, which is consistent with the current literature [21, 58]. Results also showed, as predicted, that intolerance of uncertainty was associated with the FCRI-T score. At this point, the contribution of intolerance of uncertainty on FCR etiology is not yet fully understood given that the results have varied across studies. Notably, the theoretical model validation study by Lebel et al. [27] did not find significant associations between intolerance to uncertainty and FCR. On the other hand, Curan et al. [23] showed that intolerance to uncertainty was associated with FCR in their univariable analyses, but did not predict FCR in regression analyses. However, it has been suggested that intolerance of uncertainty is an important risk factor of FCR in the majority of studies [22, 24, 25, 59]. Since cancer is characterized by a great deal of uncertainty and since a recurrence always remains possible, people with a high level of intolerance of uncertainty would have more difficulties adapting to this medical condition [1, 60]. These patients have a propensity to focus their attention on uncertain or ambiguous situations [61, 62]. Hence, even if the risk of recurrence is near to zero following preventive surgery, these women are likely to still focus on the small possibility that the cancer will return.

In addition, IES scores were significantly related to a higher FCR level. These results are consistent with the prior literature showing that individuals with more post-traumatic symptoms show higher levels of FCR [63, 64]. It is however important to note that the IES assesses intrusive thoughts, which is also an important feature of FCR [65, 66] that is also assessed by the FCRI. Avoidance, as assessed with the IES, was also significantly associated with higher levels of FCR as in previous studies [4, 5, 21, 53]. Indeed, as in patients with anxiety disorders [62, 67], patients experiencing cancer-related anxiety may choose to avoid various situations that could remind them of cancer and to use cognitive avoidance [68]. Although avoidance reduces the fear experienced and relieves patients in the short-term, in the long-term it will maintain their concerns about the possibility of a recurrence [68–70]. Finally, contrary to our expectations and the existing literature [71, 72], no significant association was observed between FCR level and quality of life, although the correlation was in the predicted direction. It suggests that other factors have a greater influence on the perceived global quality of life of these women such as social support [73], certain personality traits [74] and their physical, psychological and cognitive functioning [75]. Accordingly, quality of life was significantly related to anxiety and depression in this study.

Contrary to what was expected, results showed that the past preventive treatment received was not associated with the FCR level, even though surgery actually greatly reduces the risk of recurrence. Results also showed no difference on FCR whether patients had undergone both possible surgeries, mastectomy and preventive salpingo-oophorectomy, only one of the two or no preventive surgery at all. A first possible explanation for these results may be the absence of effect of the surgery on women’s intolerance to uncertainty, which is a fairly stable individual characteristic [76, 77]. It is also possible that women with a *BRCA1/2* genetic mutation continue to entertain maladaptive beliefs and use maladaptive coping (e.g., avoidance) strategies that maintain their FCR level despite having undergone a preventive surgery. In addition, Butow et al. [78] showed that individuals with clinical FCR have significantly more positive beliefs about worry (e.g. worry helps to prevent negative situations) and beliefs about worry being uncontrollable and dangerous, thus illustrating the importance of metacognitions on FCR. It is also possible that the low statistical power of the analysis reduced the possibility to detect significant differences. Finally, no relationship was found between the FCR score and the time since the most recent preventive surgery. FCR has been shown to be relatively stable over time, especially in those having high initial levels, even in individuals with a favorable prognosis [5, 6]. It is also possible that women who chose to undergo a preventive mastectomy and/or salpingo-oophorectomy are those who initially had higher FCR levels. Thus, even if preventive surgery was to lower FCR slightly, it is conceivable that it would still remain elevated.

Finally, study findings also showed a correlation between a higher FCR and a greater level of decisional conflict. Given that 82.0% of our sample were unaware of carrying a *BRCA1/2* gene mutation at the time of their cancer diagnosis, it is possible that the majority of women had to make a hurried decision regarding whether and what type of preventive surgery they would receive, thus limiting the time to weigh the advantages and disadvantages of each possible option. A study by Manne et al. [79] reported that the level of decisional conflict among breast cancer patients considering contralateral prophylactic mastectomy was strongly and negatively correlated with the level of preparedness to make a decision, defined as the amount of information received and satisfaction with it. Pesce et al. [80] also observed that women with unilateral and non-hereditary breast cancer who felt very confident and informed before a preventive procedure had a lower level of anxiety, less FCR and greater satisfaction with their decision 15 months after surgery. Our results also indicated a significant correlation between FCR and decisional regret. It is possible to postulate that high regrets are due, at least to some extent, to unmet expectations that preventive surgery would eliminate all FCR. If this hypothesis is confirmed, it would be advisable to inform women that they might still experience some levels of FCR after their preventive surgery and that a psychological intervention targeting FCR would then be recommended.

The present study has some strengths. First, the sample was diverse in terms of demographic (e.g., age) and clinical characteristics (e.g., mutation), thus allowing us to explore differences that could exist as a function of such variables. Finally, validated questionnaires were used to measure FCR and other psychological and quality of life constructs. This study also has limitations. First, given the absence of a population-based registry of breast cancer survivors carrying a BRCA1/2 mutation, a convenience sample was used. Participants were approached through a large email list distribution and had to contact us when interested to participate. Thus, a selection bias is possible and it is uncertain whether results are generalizable to the whole population. Indeed, it is possible that women who accepted to participate had a greater FCR level and a higher perceived risk of cancer recurrence. On the other hand, knowing that elevated anxiety often leads to behavioral avoidance, some women with high FCR may also have decided not to participate. Another limitation is the cross-sectional nature of the study. Hence, it was not possible to assess the evolution of FCR over time, how it evolved as a function of preventive surgery, nor to draw any causal inference. In addition, the statistical power of some comparative analyses was limited by the sample size. It is also important to note that recruitment for this study was conducted during the second and third waves of the COVID-19 pandemic [81]. Because of the impact that the pandemic had on the health care system (e.g., postponement of certain tests), this may have temporarily increased the participants’ anxiety about their medical condition [82, 83]. Because of all these limitations, results should be interpreted with caution and be considered preliminary.

## Conclusion

Although preliminary, findings of this study indicate that FCR is highly prevalent in women who have had breast cancer and are carriers of a *BRCA1/2* genetic mutation, even after undergoing preventive surgery, procedures that are known to significantly reduce the risk of recurrence. FCR was also associated with decisional conflicts and regrets. Together this underlines the need to offer these women psychological support that specifically targets FCR. Psychological intervention programs addressing FCR have shown positive and promising effects until now (e.g., *FORT* [84]; *Conquer Fear* [85], and our *Cognitive Behavioral Group Therapy* for FCR [25]), but the effectiveness of these interventions has yet to be specifically studied in carriers of the *BRCA1/2* genetic mutation. Future research, including population-based surveys, qualitative and longitudinal studies, would also be useful in order to better understand the role of FCR on the decision to proceed with preventive surgery, as well as the effect that surgery can have on FCR.

## Data Availability

The dataset used and analyzed during the current study is available from the corresponding author on reasonable request.

## Abbreviations

DCS: Decisional Conflict Scale
DRS: Decisional Regret Scale
QLQ-C30: EORTC Quality of Life Questionnaire
FCR: Fear of cancer recurrence
FRCI: Fear of Cancer Recurrence Inventory
FRCI-S: Severity subscale of the Fear of Cancer Recurrence Inventory
FRCI-T: Total score of the Fear of Cancer Recurrence Inventory
HADS: Hospital Anxiety and Depression Scale
HADS-A: Anxiety subscale of the Hospital Anxiety and Depression Scale
HADS-D: Depression subscale of the Hospital Anxiety and Depression Scale
IES: Impact of Event Scale
IUS: Intolerance of Uncertainty Scale
ROSE: Ressources en oncogénétique pour le soutien et l’éducation; (Oncogenetic Resources for Support and Education)
